# Bacterial Diversity in the Intestinal Mucosa of Dysbiosis Diarrhea Mice Treated with Qiweibaizhu Powder

**DOI:** 10.1155/2020/9420129

**Published:** 2020-01-07

**Authors:** Cheng-Xing Long, Hao-Qing Shao, Cheng-Yu Luo, Rong Yu, Zhou-Jin Tan

**Affiliations:** ^1^College of Traditional Chinese Medicine, Hunan University of Chinese Medicine, Changsha, 410208 Hunan Province, China; ^2^College of Mathematics and Finance, Hunan University of Humanities, Science and Technology, Loudi 417000, China; ^3^Hunan Key Laboratory of TCM Prescription and Syndromes Translational Medicine, Hunan University of Chinese Medicine, Changsha, 410208 Hunan Province, China

## Abstract

The current research tried to explore the effect of Qiweibaizhu powder (QWBZP) on the bacterial diversity and community structure of the intestinal mucosa of dysbiosis diarrhea mice and provide a scientific basis for the efficacy of QWBZP on antibiotic-induced diarrhea. A dysbiosis diarrhea mouse model was constructed with broad-spectrum antibiotics through a mixture of cephradine capsules and gentamicin sulfate (23.33 mL·kg^−1^·d^−1^). Intestinal mucosa was collected, and DNA was extracted from each group. The bacterial characteristics in intestinal mucosa were analyzed by MiSeq sequencing based on the 16S rRNA sequencing platform. There were no significant differences in alpha diversity indices among the three groups. The sample distributions in both the normal and QWBZP groups were relatively concentrated, and the distance among individuals was close. However, an opposite result was obtained in the model group. Furthermore, the composition and abundance of species were similar between the normal group and the QWBZP group at both the phylum and genus levels. After treatment with QWBZP, the abundance of *Lactobacillus* increased, and *Proteobacteria* decreased, and the Firmicutes/Bacteroidetes ratio decreased to a normal level. Our results indicate that QWBZP can help repair mucosal bacterial structure and recover mucosal microbiota. Specifically, QWBZP increased the abundance of *Lactobacillus* and *Bacteroidales* S24-7 group norank.

## 1. Introduction

The human intestine is inhabited by a large number of bacteria that adhere to the surface of the intestinal mucosa and participates in many physiological functions of the host, such as digestion, metabolism, immune regulation, energy conversion, and mucosal development and barrier maintenance [1–4]. The composition and function of the intestinal microbiota affect the health of the host [5, 6]. Usually, intestinal bacteria live in a relatively stable dynamic balanced environment. Once this balance is destroyed, the type, quantity, proportion, and location of intestinal bacteria will be disordered, resulting in dysbiosis, which will lead to pathological changes in the host [7].

The intestinal mucosa is the largest contact surface between the body environment and the intestinal cavity. It consists of mucosal epithelial cells, tight intercellular links, and bacterial membranes. It can effectively prevent the invasion of harmful substances and pathogens to maintain the stability of the internal environment [8]. Dysbiosis will cause body diarrhea as a result of the excessive increase in conditional pathogens, leading to intestinal mucosal barrier damage, shifts in intestinal bacteria and endotoxin, weakened immunity, and increased intestinal permeability [8–10]. Studies have shown that Chinese medicine has obvious advantages in the treatment of intestinal mucosal barrier dysfunction. Chinese medicine has reduced the intestinal mucosal permeability, promoted intestinal peristalsis, promoted the growth of beneficial bacteria, inhibited the growth of spoilage bacteria, stimulated the secretion of intestinal mucosa-related cells, and enhanced intestinal immunity by regulating intestinal microbiota, which improved intestinal mucosal barrier function [10]. For example, QWBZP could effectively treat dysbiosis diarrhea by improving intestinal dysbiosis and promoting the reproduction of beneficial bacteria such as *bifidobacteria* and *lactobacilli* [11, 12].

In our previous studies, we have studied the effect of QWBZP on the bacterial lactase genes, but the functional lactase function gene cannot be detected in all bacteria, only lactase-producing bacteria; however, 16S rDNA can be detected in all bacteria. Moreover, QWBZP has a good effect on intestinal structure, but it did not improve the diversity of the bacterial lactase gene in intestinal contents [13, 14]. Compared with intestinal contents, mucosa has many advantages, including completed structure, healthy immunity, relatively stable in microbiota colonization, and less affected by external factors. In this study, we aimed to investigate the effect of QWBZP on dysbiosis diarrhea from the diversity and community structure of microbiota in intestinal mucosa and provide more powerful evidence for the ability of Chinese medicine to alter the intestinal microbiota to improve intestinal mucosal barrier function. It will help develop preventions and treatments for clinical digestive diseases.

## 2. Materials and Methods

### 2.1. Animals

Eighteen one-month-old-specific pathogen-free (SPF) Kunming mice (KM) (nine males and nine females) weighing 18 g-22 g were purchased from Hunan Slaccas Jingda Laboratory Animal Company (Hunan, China) with license number SCXK (Xiang) 2016-2002. All experiments and procedures involving animals were performed according to the protocols approved by the Institutional Animal Care and Use Committee of Hunan University of Chinese Medicine.

### 2.2. Medicine

According to the Chinese Pharmacopoeia 2015, QWBZP was composed of ginseng, 6 g (Shanxi); poria cocos, 10 g (Yunan); costustoot, 6 g (Yunnan); agastache, 10 g (Guangdong); pueraria, 10 g (Hunan); *Atractylodes macrocephala*, 10 g (Zhejiang); and liquorice, 3 g (Neimong), which were purchased as prescriptions from the First Affiliated Hospital of Hunan University of Traditional Chinese Medicine. The medicine was weighed according to the above ratio and soaked for 30 min covered with cold water; the medicine was decocted with a large flame until it boiled and then with a soft flame. It was boiled twice, and the decoction time was usually 20-30 min. The liquid medicine decocted twice was mixed to make a 100% dose of a traditional decoction of QWBZP and frozen at 4°C for the following experiment [15, 16].

### 2.3. Reagents

Cephradine capsules (product batch number: 110804) and gentamicin sulfate injection (product batch number: 5120106) were purchased from Suzhou Chung-Hwa Chemical & Pharmaceutical Industrial Co., Ltd. and Yichang Renfu Pharmaceutical Co., Ltd., respectively. An antibiotic mixture was prepared at a concentration of 62.5 g L^−1^ with sterile saline according to the ratio of 1 : 2 (i.e., 6 gentamicin (2 mL/branch) + 3 cephalosporins (0.25 g/grain)) and stored at 4°C [16, 17]. Protease K, Tris-saturated phenol-chloroform-isoamyl alcohol (25 : 24 : 1), lysozyme, and TE buffer were purchased from Beijing Ding Guo Biotechnology Co., Ltd. Other reagents were prepared in the lab.

### 2.4. Methods

After 2 days of adaptive feeding, eighteen one-month-old SPF KM mice (equal numbers of males and females) were randomly divided into three groups, six mice (three males and three females) per group: normal group (mn), model group (then called natural recovery group, mm), and treatment group (mq). To induce diarrhea, mice in both the model group and treatment group were administered an antibiotic mixture composed of gentamicin sulfate and cephradine (23.33 mL·kg^−1^·d^−1^). Correspondingly, mice in the normal group were gavaged with sterile water, 0.35 mL twice a day for 5 days [11, 16]. When diarrhea symptoms (declined activity, arched back trembling, specifically watery stool, curled up, and poor appetite) were induced, the mice in the treatment group were administered the QWBZP decoction at a dosage of 0.16 g·kg^−1^·d^−1^ for 3 days [12, 16]. Mice were sacrificed using cervical vertebra dislocation on a sterile operation platform. Then, intestinal mucosa was scraped with cover slips, and 2 times the weight of saline was added after squeezing out the chymus, cutting open the intestinal tract and cleaning the intestinal wall with saline. Finally, intestinal mucosa samples from two mice (one male and one female) in every group were selected and immediately frozen at 4°C for the following experiment [16, 18].

### 2.5. Metagenome Extraction

According to our previous report, metagenome DNA from the intestinal mucosa microorganisms was extracted by the following protocols [19]. A total of 2.0 g of intestinal mucosa were collected in a sterile environment, placed in a germ-free centrifuge tube, and homogenized in 5 mL of 0.1 mol L^−1^ phosphate buffered solution (PBS), and centrifuged at 200 × g for 2 min. After washing twice with PBS, the supernatant was transferred into new germ-free tubes and centrifuged at 10,000 × g for 8 min. Then, the new sediments were gathered; washed once with PBS, twice with acetone, and three times with PBS; and resuspended in 4 mL of TE buffer. After sample pretreatment, 500 *μ*L of suspension, 20 *μ*L of lysozyme, 5 *μ*L of proteinase K, and 45 *μ*L of TE buffer were added to 1.5-mL germ-free EP tubes and homogenized. Samples were incubated at 37°C for 30 min and mixed with 30 *μ*L of 10% SDS, followed by incubation at 37°C for 40 min, with vortexing once every 10 min. Afterwards, 80 *μ*L of hexadecyl trimethyl ammonium bromide (CTAB)/NaCl and 100 *μ*L of 5 mol L^−1^ NaCl were added and mixed well. The mixture was vortexed at 65°C for 10 min. An equal volume of Tris-saturated phenol-chloroform-isoamyl alcohol (25 : 24 : 1) was then added to the sample, mixed well and centrifuged at 10,000 × g for 3 min. The supernatant was transferred into new germ-free tubes, mixed with an equal volume of chloroform-isoamyl alcohol (24 : 1), and centrifuged at 10,000 × g for 3 min. The supernatant was transferred to fresh germ-free tubes, and an equal volume of chloroform-isoamyl alcohol (24 : 1) was added and mixed well again. The supernatant was transferred to fresh germ-free tubes after centrifugation at 10,000 × g for 3 min, 10^−1^ volume of 3 mol L^−1^ sodium acetate and double volume of absolute ethyl alcohol were added and precipitated at -20°C for approximately12 h. Samples were centrifuged at 10,000 × g for 3 min. The acquired sediment was washed with 70% ethanol, dried, and eventually dissolved in 50 *μ*L of TE buffer for DNA metagenome extraction.

### 2.6. PCR Amplification and MiSeq Metagenome Sequencing

The V3+V4 variable region of bacterial 16S rDNA was amplified using the extracted DNA as a template. The primers used for amplification were 338F (5′-ACTCCTACGGGAGGCAGCA-3′) and 806R (5′-GGACTACHVGGGTWTCTAAT-3′). The PCR mixtures (20 *μ*L) contained 2.0 *μ*L of 10 × buffer, 2.0 *μ*L of 2.5 mmol/L dNTPs, 0.8 *μ*L of forward primer (5 *μ*mol/L), 0.8 *μ*L of reverse primer (5 *μ*mol/L), 0.2 *μ*L of Taq Polymerase, 0.2 *μ*L of BSA, 10 *μ*L of template DNA, and 4 *μ*L of ddH_2_O. PCR conditions were as follows: initial denaturation at 95°C for 3 min, followed by 29 cycles at 95°C for 30 s, annealing at 55°C for 30 s, and extension at 72°C for 45 s, and then 72°C for 10 min [20]. The PCR products were examined by 2% agarose gel electrophoresis, purified using the AxyPred Gel Extraction Kit (Axygen, Scientific Inc., Union City, CA, USA), and quantified using QuantiFluor™-ST (Promega). The PCR products were then sequenced by the Illumina MiSeq sequencing platform (Illumina, San Diego, CA, USA). MiSeq metagenomic sequencing was completed by Wuhan Fraser Genetic Information Co., Ltd.

### 2.7. Bioinformatics and Statistical Analysis

Bacterial diversity indices (including Chao1, ACE, Simpson, and Shannon index) in the intestinal mucosa were measured by MOTHUR (version v.1.30.1, http://mothur.org/) based on the operational taxonomic units (OTUs) [21]. Principle component analysis (PCA), principal coordinate analysis (PCoA), nonmetric multidimensional scaling (NMDS), and LEfSe analysis were conducted with the R package (http://www.R-project.org/) to analyze the main distribution characteristics and the similarity of community samples [22–25]. SPSS 24.0 software (IBM Corp, Armonk, NY, USA) was used to analyze the statistical data. Independent sample *t* test with *P* < 0.05 or *P* < 0.01 was applied to compare the statistical significance of differences.

## 3. Results

### 3.1. Effect of QWBZP on Bacterial OTU Number in the Intestinal Mucosa of Dysbiosis Diarrhea Mice

**Figure 1:**
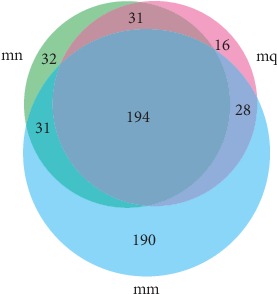
Effects of QWBZP on the number of intestinal mucosa bacterial OTUs. The Venn diagram represents the number of shared and unique OTUs for the three groups, mn for the normal group, mm for the model group, and mq for the treatment group.

Using the USEARCH (vsesion7.1) software platform, the sequences were subjected to OTU clustering of nonrepetitive sequences (excluding single sequences) according to 97% similarity. The analysis results are shown in Figure 1. There were 288, 443, and 269 OTUs found in the normal group, natural recovery group, and treatment group, respectively. Among them, 194 were identical. This indicates that the antibiotic increased the number of bacterial OTUs in the intestinal mucosa and increased the bacterial species in mice. After treatment with QWBZP, the number of bacterial species decreased close to normal levels.

### 3.2. Effect of QWBZP on Bacterial Diversity in the Intestinal Mucosa of Dysbacterial Diarrhea Mice

#### 3.2.1. Alpha Diversity Analysis

**Figure 2:**
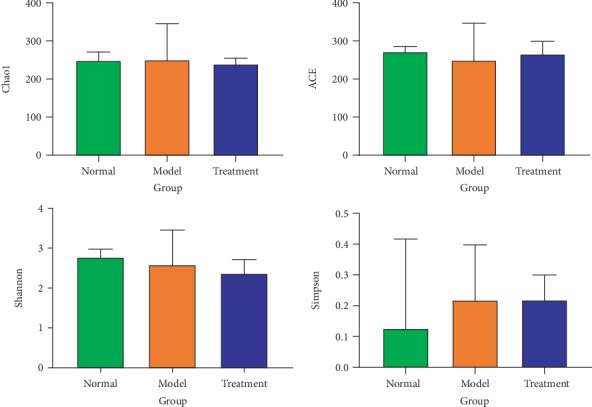
Effects of QWBZP on intestinal mucosa bacterial diversity. Alpha diversity indices can reflect the abundance and diversity of microbial communities. Green, orange, and blue represent the normal, model, and treatment groups, respectively.

Alpha diversity analysis can reflect the abundance and diversity of microbial communities. Chao1 and ACE are often used to estimate the total number of species. The larger the index value is, the larger the total number of species. Simpson and Shannon are usually used to quantitatively describe the diversity of organisms in a region. The smaller the Simpson index and the larger the Shannon index, the higher the community diversity is. The results showed that the ACE, Chao1, Simpson, and Shannon indices in the natural recovery group are very close to those of the treatment group, and the ACE, Chao1, and Shannon indices are slightly lower; however, the Simpson index of the treatment group is slightly higher than that of the normal group, and there are no significant differences (Figure 2). It is suggested that the effect of QWBZP on the bacterial diversity in intestinal mucosa is similar to natural recovery, and it does not increase its diversity.

#### 3.2.2. Beta Diversity Analysis

**Figure 3:**
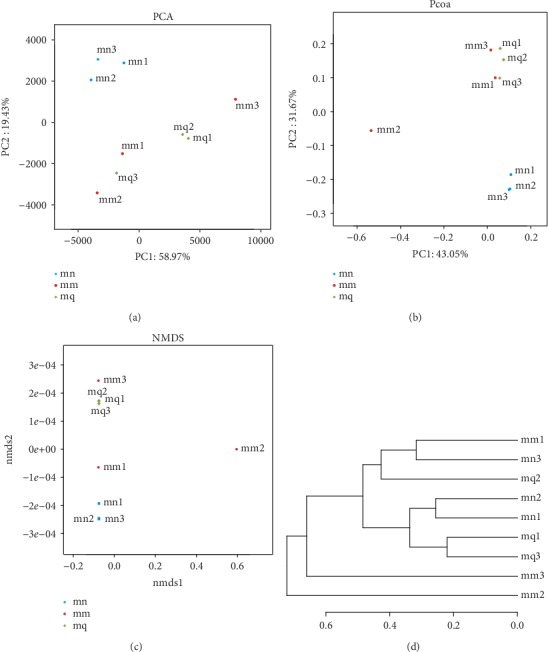
Effects of QWBZP on the intestinal mucosa bacterial structure. Beta diversity analysis was used to analyze the bacterial community structure. Blue, red, and green represent the normal, model, and treatment groups, respectively: (a) PCA analysis, (b) PCoA analysis, (c) NMDS analysis, and (d) Hierarchical cluster analysis.

PCA can reflect the differences and distances between samples by analyzing the OTU (97% similarity) composition of different samples, which reflects the differences in multiple sets of data on a two-dimensional coordinate graph using variance decomposition. The more similar the sample composition is, the closer the distance in the PCA diagram. The percentage contributing to the variation in PC1 and PC2 was 96.67% and 1.71%, respectively (Figure 3(a)). Compared with the normal group, samples in the natural recovery group were relatively scattered, and the distance between the two groups was great, indicating that antibiotics had a significant impact on the bacterial structure in the intestinal mucosa. After treatment with QWBZP, samples mq1 and mq2 were concentrated, but mq3 was relatively dispersed, which may be related to individual differences. These findings indicate that QWBZP can effectively restore bacterial structure in the intestinal mucosa of dysbiosis diarrhea mice. Moreover, we obtained the same result in PCoA and NMDS (Figures 3(b) and 3(c)).

To further investigate the similarity of bacterial genes, HC was used based on the UniFrac distance matrix. As shown in Figure 3(d), mn1, mn2, mn3, mm1, and mq2 are clustered into a category, and mm2 and mm3 are clustered separately. This indicates that the similarity between normal group samples is high, indicating that antibiotics may destroy the mucosal bacterial structure of normal mice. After treatment with QWBZP, mq1 and mq3 can be well clustered with mn1 and mn2, mn3 and mq2 are clustered into a category. This indicates that the difference in bacterial structure is small and the similarity is high between the treatment and normal groups, suggesting that the treatment effect is good. However, mm1 and mn3 are clustered together, which may be related to individual differences in mice.

### 3.3. Effect of QWBZP on the Community Composition of the Intestinal Mucosa Bacteria in Dysbiosis Diarrhea Mice

#### 3.3.1. Effect of QWBZP on the Composition of Intestinal Mucosa Bacteria at the Phylum Level

**Figure 4:**
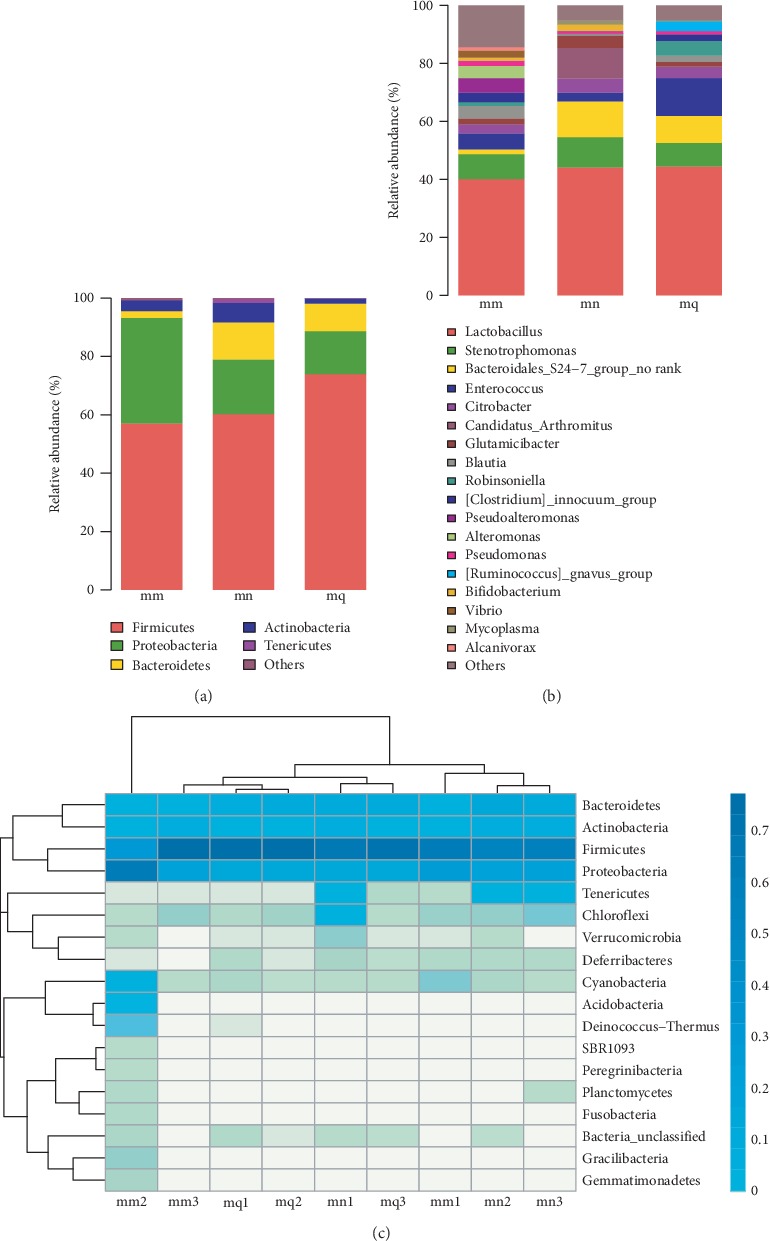
Effects of QWBZP on intestinal mucosa bacterial abundance. Relative abundance of the intestinal mucosa bacteria (a) at the phylum level and (b) at the genus level. (c) Heatmap analysis of the intestinal mucosa bacteria at the phylum level. Mn, mm, and mq represent the normal, model, and treatment groups, respectively.

In general, the intestinal mucosa bacteria mainly originated from Firmicutes, Proteobacteria, Bacteroidetes, Actinobacteria, and Tenericutes. Firmicutes was the most abundant. Among the detected bacteria, Acidobacteria, Gracilibacteria, Gemmatimonadetes, and Deinococcus-Thermus were only found in the natural recovery group. It is not difficult to see from Figure 4(a) that the abundances of Firmicutes, Bacteroidetes, Actinobacteria, and Tenericutes in the natural recovery group were lower than those in the normal group, except for Proteobacteria. Additionally, there were significant differences in Actinobacteria (*P* = 0.027), Bacteroidetes (*P* = 0.001), and Tenerictutes (*P* = 0.002). After treatment with QWBZP, the abundance of Firmicutes, Bacteroidetes, and Tenericutes increased, and the abundance of Proteobacteria and Actinobacteria decreased. Furthermore, the abundance of Firmicutes was higher, while the abundances of Proteobacteria and Actinobacteria were lower in the treatment group than those in the normal group, but there was no significant difference.

To confirm our findings, a heat map analysis provided by the R package was used. A heat map can visually represent the size of a data value in a defined shade of color. Horizontal represents clustering of abundance similarity between samples, and vertical represents clustering of abundance similarity between species. High-abundance and low-abundance species can be aggregated by clustering, and the similarity and difference of community composition at each classification level are reflected by color gradient and similarity. In the detected bacteria at the phylum level, bacteria were clearly separated by their relative abundance (Figure 4(b)).

#### 3.3.2. Effect of QWBZP on the Composition of Intestinal Mucosa Bacteria at the Genus Level

Among the detected bacteria at the genus level, *Lactobacillus* (44.16%) was the dominant species in the normal group, followed by *Bacteroidales*_S24-7_group_norank (12.33%), *Candidatus arthromitus* (10.61%), and *Stenotrophomonas* (10.41%). However, the dominant species in the natural recovery group were *Lactobacillus* (40.05%), *Stenotrophomonas* (8.66%), *Enterococcus* (5.53%), and *Pseudoalteromonas* (5.05%). The dominant species in the treatment group were *Lactobacillus* (44.51%), *Enterococcus* (12.97%), *Bacteroidales*_S24-7_group_norank (9.32%), and *Stenotrophomonas* (8.10%) (Figure 4(c)). Among the six dominant genera, *Bacteroidales*_S24-7_group_norank and *Candidatus arthromitus* in the natural recovery group were significantly lower than those in the normal group (*P* < 0.01); compared with the normal group, *Enterococcus* was increased and *Lactobacillus, Pseudoalteromonas*, and *Stenotrophomonas* were decreased, but there was no significant difference. After treatment with QWBZP, *Lactobacillus* and *Bacteroidales*_S24-7 increased, reaching the normal group level; *Stenotrophomonas* and *Candidatus arthromitus* were similar to the natural recovery group but lower than the normal group. In addition, *Enterococcus* continued to increase, which was significantly different from the natural recovery group and the normal group (*P* < 0.05).

## 4. Discussion

### 4.1. QWBZP Helps to Restore Bacterial Diversity in Intestinal Mucosa

In the species diversity analysis, there are two factors, namely, richness and uniformity. In this study, the Chao1, ACE, and Shannon indices were the largest, and the Simpson index was the smallest in the normal group. To some extent, it reflected the richness and complexity of intestinal mucosal bacteria in normal mice. The diversity indices in the treatment group were very close to those in the natural recovery group, and there was no difference from the normal group, which is consistent with our previous findings [20]. The possible reason may be closely related to the strong self-regulating effect in mucosal bacteria; the mucosal microbiota can be naturally recovered after short-term antibiotic intervention because of its strong resilience. The number of OTUs (443) in the natural recovery group was significantly higher than that those in the normal group (288) and QWBZP group (269), and the number of OTUs in the QWBZP group was close than that in the normal group, indicating that QWBZP could help restore the mucosal bacterial diversity to a normal level. This result contrasts with previous studies in which antibiotic intervention and QWBZP treatment have a great impact on bacterial diversity in the intestinal contents [26]. It may be related to several factors, including short treatment time, the priority of QWBZP to restore the microbiota in intestinal contents, and different components of intestinal microbiota between contents and mucosa.

### 4.2. QWBZP Regulates the Bacterial Abundance in Intestinal Mucosa

From the species abundance analysis, the composition and abundance of species was similar between the normal group and the QWBZP group at both the phylum and genus levels. The natural recovery group increased the abundance of many unclassified bacteria. In specific taxonomic units, antibiotics increased the bacterial phyla abundance of Acidobacteria, Gracilibacteria, Gemmatimonadetes, and Deinococcus-Thermus and the bacterial genera abundance of *Acidobacteria*_norank, *Aestuariibacter*, *Alteromonas*, *Aureicoccus*, *BD*1-7_clade, *Halomonas*, *Hoeflea*, *Labrenzia*, *Marinobacter*, *Pseudoalteromonas*, *Rhodobacteraceae*_unclassified, *Ruegeria*, *Shimia*, *Thalassobius*, and *Vibrio*. This result indicates that antibiotics promoted the increase in many pathogenic bacteria to a certain extent and QWBZP contributed to the recovery of bacterial abundance in intestinal mucosa.

The increasing number of Proteobacteria was a diagnostic sign of dysbiosis and disease risk, and the ratio of Firmicutes and Bacteroidetes (*F*/*B* value) was commonly used to measure intestinal homeostasis [27]. *Lactobacillus* inhibits intestinal pathogenic bacteria, promotes the development of T cells, and enhances cellular immunity [28]. In this study, after treatment with QWBZP, *Lactobacillus* increased, *Proteobacteria* decreased, and the *F*/*B* ratio decreased to a normal level. Moreover, *Enterococcus* continued to increase, which was significantly different from the natural recovery group and the normal group (*P* < 0.05). These results indicated that QWBZP helped to recover the main microbiota and reach a new balance in intestinal mucosa. In addition, the proliferation of *Lactobacillus* promotes the repair of intestinal mucosa, and the increase in *Enterococcus* may be related to the resistance of antibiotics, such as gentamicin.

### 4.3. QWBZP Effectively Repaired the Bacterial Community Structure in the Intestinal Mucosa

PCA was used to analyze the bacterial community structure. From our results, the distribution was relatively concentrated, and the sample distance was close both in the QWBZP and normal groups, suggesting a high similarity in bacterial community structure. In the natural recovery group, the sample distribution was relatively scattered, and the sample distance was far from both the QWBZP group and normal group, indicating poor similarity. We obtained the same result in subsequent PCoA and NMDS analysis. The findings showed that antibiotics destroyed the bacterial structure of the intestinal mucosa in normal mice. These results are consistent with our previous study that antibiotics significantly altered the community structure of intestinal mucosal bacteria [20]. In addition, samples in both the normal and QWBZP groups were more concentrated, and the similarity in structure was high, suggesting that QWBZP could help restore the bacterial community structure in intestinal mucosa to a normal level. We believe that it is related to the effective repair effect of QWBZP on the intestinal mucosal bacterial structure in mice with dysbiosis diarrhea [13, 29].

In conclusion, antibiotics destroyed the intestinal mucosa bacterial structure in mice and, to a certain extent, stimulated the increase in their diversity. After treatment with QWBZP, bacteria were effectively restored, the structure was effectively repaired, and microbiota reached a new balance in intestinal mucosa. Our study suggests that the probable efficacy of QWBZP on dysbiosis diarrhea mice was achieved through the effective recovery of the structure and microbiota in intestinal mucosa.

## Data Availability

The data used to support the findings of this study are available from the corresponding author upon request.
